# Synchronous anal canal carcinoma in a heterosexual couple

**DOI:** 10.1186/s12885-018-4785-8

**Published:** 2018-09-10

**Authors:** Lucas C. Mendez, Eugene Hsieh, Craig C. Earle, Shun Wong

**Affiliations:** 1 Department of Radiation Oncology, Sunnybrook Health Sciences Centre T-Wing, University of Toronto, 2075 Bayview Ave, Toronto, ON M4N 3M5 Canada; 2 Division of Anatomic Pathology, Department of Laboratory Medicine and Molecular Diagnostics, Sunnybrook Health Sciences Centre, Toronto, Canada; 3 Department of Laboratory Medicine and Pathobiology, University of Toronto, Toronto, Canada; 4 Division of Medical Oncology, Department of Medicine, Sunnybrook Health Sciences Centre, University of Toronto, Toronto, Canada; 5 Institute of Clinical Evaluative Sciences, Toronto, Canada

**Keywords:** HPV infection, Anal canal cancer, Heterosexuality

## Abstract

**Background:**

Sexually transmitted Human Papilloma Virus (HPV) infection is a known risk factor for cancer of the anal canal in both men and women.

**Case presentation:**

We describe a report of synchronous carcinoma of the anal canal in a heterosexual couple. High risk type 16 HPV DNA was detected in both tumors.

**Conclusion:**

Longstanding sexual partners may share risk of HPV-associated anal canal cancer.

## Background

Over 8,000 new cases of cancer of the anal canal are expected in the United States in 2017, with 2/3 of them in women [1]. The incidence of anal cancer continues to grow in both sexes, with a more pronounced increase in the male population [2]. Infection by the human papilloma virus (HPV) is strongly associated with anal cancer, with almost all cases associated with this virus [3]. Sexually transmitted disease, immunosuppression such as HIV infection, tobacco use and previous history of cervical, vaginal or vulvar cancer are known risk factors for the disease [4–6]. Men who have sex with men are at higher risk of developing anal canal cancer than heterosexual men [7]. Here we reported synchronous presentation of squamous cell carcinoma of the anal canal in a heterosexual couple.

## Case presentation

### Case 1

**Fig. 1:**
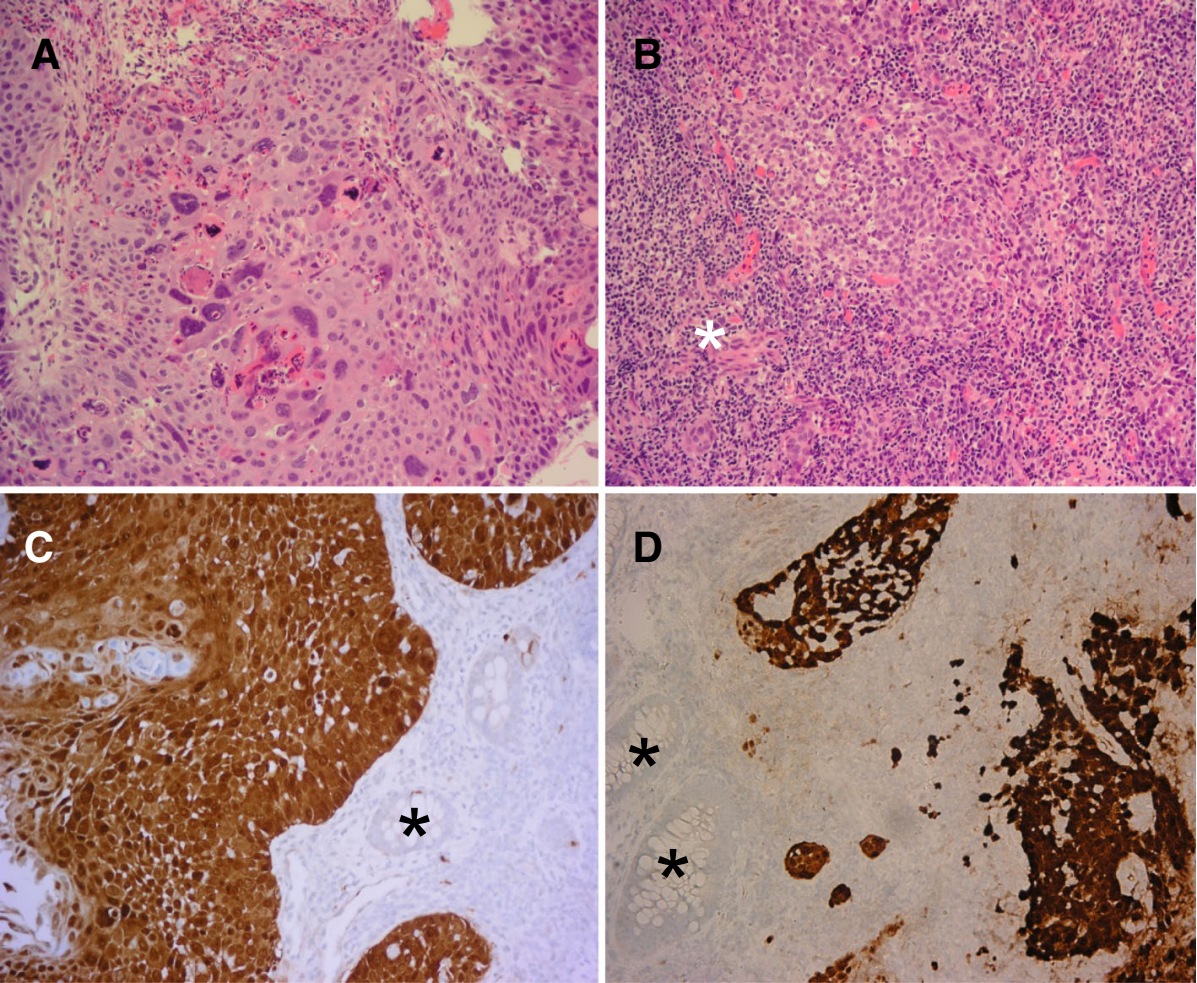
The biopsy of the anal lesion of the female revealed a well-differentiated squamous cell carcinoma (**a**), and the male had a poorly differentiated squamous cell carcinoma (**b**) with a prominent lymphoid infiltrate (*), hematoxylin and eosin staining. Both lesions (**c**, **d**) demonstrate strong and diffuse p16 immunoreactivity (brown) and there is no p16 expression in the adjacent rectal glands (*). Magnification X 200

**Fig. 2:**
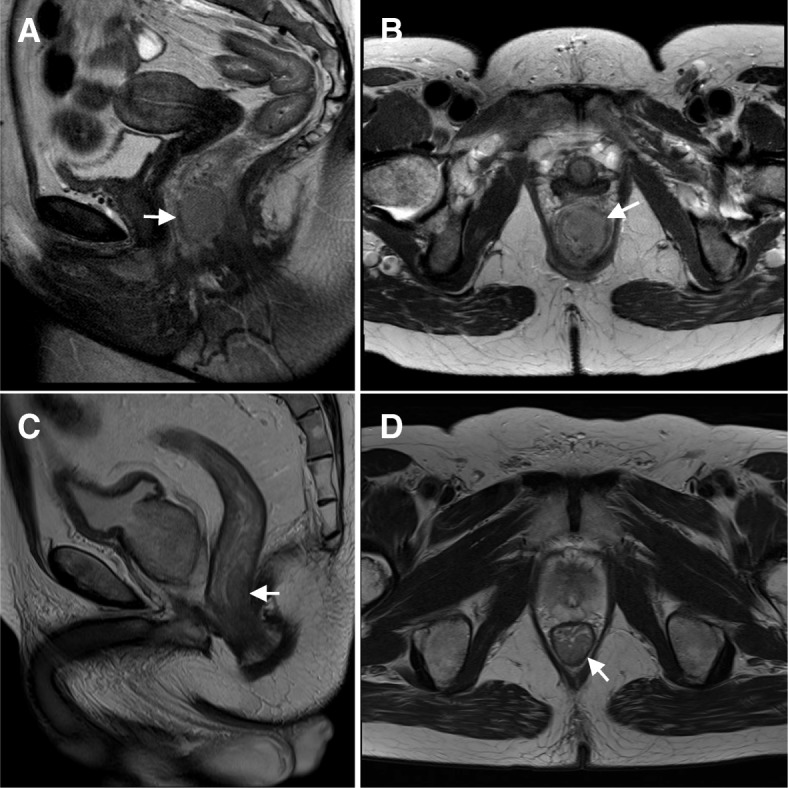
T2-weighted MRI of the pelvis of the female case shows a 3-cm lesion in the anal canal extending into the anorectal junction (**a**. sagittal, **b**. axial). MRI of the male case also demonstrates a T2 lesion measuring about 3 cm beginning at 2 cm from the anal verge (**c**. sagittal, **d**. axial)

A 60-year-old woman sought medical attention after a 2-month history of minor rectal bleeding and an anal nodule. On physical examination, a 3-cm mobile anterior ulcerative mass in the anal canal was palpable, beginning at 1 cm from the anal verge with no extension to the anorectal junction. No nodes were appreciated in the inguinal regions. A biopsy revealed an invasive squamous cell carcinoma, well-differentiated (Fig. 1a). Staging computerized tomography (CT) of the thorax/abdomen/pelvis did not show any lymphadenopathy or distant metastatic disease. Pelvic magnetic resonance imaging (MRI) demonstrated a 3-cm mass in the anal canal extending to the anorectal junction (Fig. 2a and b). There was no pelvic or inguinal lymphadenopathy. Her laboratory investigations including HIV-1 and HIV-2 serology were negative. A recent Papanicolau smear of the cervix was reported to be negative for intraepithelial lesion or malignancy.

The patient had a past medical history remarkable for an in-situ cervical carcinoma treated with laser therapy over 20 years ago with no subsequent recurrence. She also underwent a right salpingo-oophorectomy for an endometriotic cyst of the right ovary 8 years prior to her anal canal cancer diagnosis. She is a life-time non-smoker and social alcohol drinker. There was no past history of any autoimmune disorders.

For her T2N0M0 anal cancer, the patient underwent a course of chemo-radiotherapy as per institutional protocol. The total dose was 54 Gy in 30 daily fractions to the primary and elective nodal irradiation consisted of 36 Gy in 20 fractions to the inguinal/femoral, mesorectal, presacral and external/internal iliac nodal regions. The radiation treatment was delivered using volumetric modulated arch therapy (VMAT) technique. The chemotherapy regimen consisted of mitomycin C (10 mg/m^2^ on day 1) and infusional 5-fluorouracil (1000 mg/m^2^/day for 4 days), given concurrently on week 1 and week 5 of radiation. The patient developed RTOG grade 3 skin and perineal reactions. She had an episode of fever without neutropenia managed by oral antibiotics.

### Case 2

While the female was undergoing chemoradiation, her husband, with whom she has been married for over 30 years, requested a screening colonoscopy during his regular urological follow-up. The investigation revealed an anal mass that was biopsied and demonstrated a poorly-differentiated squamous cell carcinoma with prominent lymphoid infiltration (Fig. 1b). This 63 year-old man was largely asymptomatic. After diagnosis, he was referred to the same cancer centre where his wife received oncological care. On physical examination, an ulcerative exophytic mass was seen, measuring approximately 3 cm in maximum dimension, located over the posterior third of the anal canal at 2 cm from the verge. The mass extended to the anorectal junction. No inguinal nodes were clinically suspicious. Staging CT and MRI revealed at least four suspicious mesorectal nodes and a 1.8-cm left external iliac lymph node. There was no evidence of distant metastatic disease. On MRI, the maximum tumor dimension of the primary was 3 cm (Fig. 2c and d). Similar to his wife, this man’s HIV serology was negative. He was a non-smoker. There was no previous history of sexually transmissible disease, and he denied any receptive anal intercourse or sex with men. He was on tamsulosin 0.4 mg and dutasteride 0.5 mg for benign prostatic hyperplasia.

This patient was treated with the same institutional protocol of chemoradiation for his T2 N2 anal cancer with a total dose of 54 Gy to the primary and the involved nodes plus concurrent chemotherapy with 5-fluorouracil and mitomycin C. The elective nodal irradiation dose was 36 Gy. The patient developed neutropenia and thrombocytopenia, diarrhea, oral mucositis and the usual perianal/perineal skin reaction.

The immunohistochemical profiles of both the wife’s and husband’s lesions were similar. Both tumours demonstrated diffuse and strong immunoreactivity for p16 (Fig. 1c-d) suggesting high-risk HPV infection [8] and neither exhibited p53 overexpression or p53 loss. PCR for HPV genotype was performed using the Cobas 4800 HPV Test System [9]. HPV16 DNA was detected in both lesions. The two lesions were negative for HPV18 and also negative for HPV31, 33, 35, 39, 45, 51, 52, 56, 58, 59, 66 and 68.

## Discussion and conclusion

Two-thirds of anal canal cancer cases are seen in females and the incidence among men is about 20 times higher in men who have sex with men [5]. Therefore, little attention has been focused on heterosexual men in regard to cancer in the anal region. However, up to 12–24% of heterosexual men have been reported to harbor HPV infection in the anal canal [10, 11]. It is expected that some of these patients, as described here, may develop anal carcinoma.

This report brings to light two interesting points. First, the route of HPV transmission to the anal canal in men who do not have receptive anal sex is not yet fully understood. One possible route of transmission is self-contamination from genital or perianal sites that may occur with either sexual or non-sexual behavior. An association between genital and anal canal HPV co-infection has been described [4]. In fact, multiple overlapping risk factors, such as increasing number of female sex partners and short duration of the relationship have been reported as risk factors for both penile and anal cancers [10, 12]. Non-sexual routes of transmission may also explain anal canal infection in men that do not have sex with men, especially for patients without genital HPV infection. Previous studies have identified HPV-DNA in fingers [13], various objects such as gloves and biopsy forceps [14], suggesting that multiple items could serve as a potential vehicle for HPV transmission. It is not clear, however, if exposure to these fomites could result in actual infection. This concept also strengthens the idea of self-contamination. Finally, under reporting of receptive anal intercourse is another possible explanation as patients may refuse to disclose this information due to embarrassment and stigma.

The second learning point illustrated by this report of the 2 cases relates to the theoretical higher risk of development of HPV-related cancers in partners of patients with cancers associated with this virus. Recently, a systematic review suggested a 2–3 fold increase risk of HPV-related cancer in spouses of patients with previous HPV-related malignancies [15]. Similar to the two cases described here, Andrews et al. provided an example of this notion in a report of two heterosexual couples diagnosed with HPV-related oropharyngeal carcinomas within a time frame of one year [16]. Interestingly, both husbands and wives in this report were also found to have HPV16-associated tumors. We did not perform deep sequencing of the HPV16 genome in the couple in the present study. Although sequence concordance may lend further evidence for partner-to-partner transmission, the HPV genome is known to exhibit genetic diversity generated through interactions with host cell DNA-editing enzymes [17]. It may thus be difficult to prove partner-to-partner transmission definitively.

Anal canal cancer is an uncommon entity that most frequently affects women or men who have sex with men. This report suggests that men who have sex with women may be at risk of cancer of the anal region. In conclusion, both male and female partners of patients with HPV-related cancers are at increased risk of malignancies associated with this virus, including carcinoma of the anal canal.

## Abbreviations

CT: Computerized tomography
HIV: Human immunodeficiency virus
HPV: Human papilloma virus
MRI: Magnetic resonance imaging
VMAT: Volumetric modulated arch therapy
